# Non-Chemical Distant Cellular Interactions as a potential confounder of cell biology experiments

**DOI:** 10.3389/fphys.2014.00405

**Published:** 2014-10-17

**Authors:** Ashkan Farhadi

**Affiliations:** Digestive Disease Center, Memorial Care Medical GroupCosta Mesa, CA, USA

**Keywords:** signal transduction, communication, electromagnetic wave, placebo, placebo effect, confounding factors, cell biology, NCDCI

## Abstract

Distant cells can communicate with each other through a variety of methods. Two such methods involve electrical and/or chemical mechanisms. Non-chemical, distant cellular interactions may be another method of communication that cells can use to modify the behavior of other cells that are mechanically separated. Moreover, non-chemical, distant cellular interactions may explain some cases of confounding effects in Cell Biology experiments. In this article, we review non-chemical, distant cellular interactions studies to try to shed light on the mechanisms in this highly unconventional field of cell biology. Despite the existence of several theories that try to explain the mechanism of non-chemical, distant cellular interactions, this phenomenon is still speculative. Among candidate mechanisms, electromagnetic waves appear to have the most experimental support. In this brief article, we try to answer a few key questions that may further clarify this mechanism.

## Can cells detect and respond to electromagnetic waves?

There is no question that cells can be affected by electromagnetic waves (EMW) over a wide range of electromagnetic frequencies. This phenomenon is not just limited to specialized, light-detecting cells in our body such as retinal, and pineal cells or to cellular damage that is caused by the absorption of energy by the tissue (Mullins et al., 1999). Moreover, several reports suggested that the effects of EMW have the characteristics of receptor-mediated interactions (Albrecht-Buehler, 1981, 1991, 1994, 2000; Mullins et al., 1999). Albrechet-Buehler proposed that centrosomes are infrared detectors (cell eyes) and that microtubules are cables carrying signals between subcellular organelles (cell nerves). He observed that cultured cells move toward infrared light (Albrecht-Buehler, 1981, 1994). Indeed, doses of EMW that are not physically damaging to cells can affect several cellular functions including cellular proliferation, differentiation (Lisi et al., 2006, 2008; Foletti et al., 2009), apoptosis (Santini et al., 2005), DNA synthesis (Litovitz et al., 1994), RNA transcription (Goodman et al., 1983), protein expression (Goodman and Henderson, 1988) and many other cellular functions. In a recent review by Prasad et al. and our own review on electromagnetic-based cellular interactions, the different perspectives regarding the effect of EMW on cells at various levels of cell function are discussed (Cifra et al., 2011; Prasad et al., 2014).

## Can cells generate electromagnetic waves?

Again, there is no doubt that biological systems can actively generate and emit EMW. Scientists have reported various forms of emitted EMW since the early decades of the last century (Cifra et al., 2011). These studies can be traced back to as early as 1916, when descriptions of various forms of emitted EMW appeared in works by Scheminzky (1916) and later, regarding mitogenic radiation, by Gurwitsch (1923). Indeed, a growing number of experiments have shown that biological systems are capable of generating and emitting biophotons i.e., ultraweak photon emission (UPE) (Rahnama et al., 2011; Cifra and Pospíšil, 2014). The question remains as to whether UPE is specific and/or purposeful. The finding of unique patterns of UPE emitted from biological systems during specific phases of the cell cycle (Konev et al., 1966; Quickenden and Que Hee, 1976) suggests that UPE is specific and that UPE could have a role in non-chemical, distant cellular interactions (NCDCI), rather than being a random or spontaneous event. However, whether UPE serves any particular purpose in a specific cell function such as cell metabolism or proliferation is a question that remains to be answered. There have been several efforts to analyze the spectral details of UPE in order to further establish the role of UPE in a biological system (Rastogi and Pospíšil, 2012; van Wijk et al., 2013; Ives et al., 2014). In particular, a recent study using electron paramagnetic resonance (EPR) spectroscopy that allows an enhancement in spontaneous UPE in biological samples has enabled us to further analyze the UPE emission spectrum and use that as an indicator for specific cellular metabolism (Rastogi and Pospíšil, 2012). More research in this field will most likely provide us with newer technologies to identify the spectral analysis of these waves and guide us toward recognizing the specific function of these waves.

## Can cells use electromagnetic messages to communicate?

NCDCI are the best explanation for the synchrony between physically separated biological systems. Research findings by our lab and by others have shown that cells can communicate with other cells that are physically separated by a physical “barrier” (Kaznacheev et al., 1980; Albrecht-Buehler, 1991; Farhadi et al., 2007; Fels, 2009; Rossi et al., 2011; Chaban et al., 2013; Scholkmann et al., 2013). This barrier typically prevents any chemical or electrical communication between distant cells, and barriers can be modulated to further explore the boundaries of NCDCI (Rossi et al., 2011). These experiments may vary in design, but generally speaking, the experimental cells that are being exposed directly to an intervention are called “inducers.” In contrast to “inducer” cells, there is another group of cells that are called “detectors.” Detector cells are actually a negative control group since they are not being exposed to the intervention. However, these cells are kept within the proximity of the inducer cells while they are mechanically separated from the inducer cells by a physical barrier. There is another set of negative control cells in these studies called the non-detector control. This group is similar to the “detector” group in the sense that it is not exposed to the intervention. However, this group differs from the “detector” cells in the sense that the non-detector control is physically isolated from the “inducer” cells by placing them in a different part of the lab or in an adjacent lab. In these experiments, it has been found that the intervention-induced changes are not only observed in the inducer cells. Parallel changes can be observed in detector cells as well. However, no effect is observed in non-detector control cells. The fact that effects are observed in detector cells but not in non-detector control cells strongly suggests the existence of NCDCI.

The mechanism responsible for NCDCI is not limited to interactions at the cellular level. A similar phenomenon has been reported at the level of whole plants, primitive biosystems (such as insects), and other biosystems (Burlakov et al., 2000; Cifra et al., 2011). This method of communication could also exist at intracellular, inter-organelle, or intra-organelle levels (Havelka et al., 2011). Even though EMW seems to be the best candidate for the source of NCDCI, the nature of the signaling is still poorly understood and needs further exploration. The generator or receiver of the EMW could be a cellular biochemical reaction, macromolecular structure or a sub-cellular organelle. There is no solid scientific data to show which range or types of EMW are being used. Furthermore, a theoretical paper raised concern regarding the viability of EMW as a method of intercellular communication due to weak intensity of the emission and an unfavorable signal-to-noise ratio for these waves in natural conditions (Kučera and Cifra, 2013). Considering these parameters, the most plausible form of EMW that could be considered as the signaling method of interest is a modulated electromagnetic waveform data package that is capable of transferring digital information.

## Can NCDCI account for a confounder in controlled experiments?

The placebo effect is a significant confounder in many clinical studies with human subjects and some animal studies. Therefore, almost all current clinical trials include a placebo arm in one way or another (Miller and Rosenstein, 2006; Muñana et al., 2010). Even though a placebo effect is not a concern in cell-biology experiments, controlling all experimental variables is still very important. For this reason, all biological as well as biochemical experiments are done as controlled experiments. In most cell-biology experiments, the experimental samples are exposed to an intervention and the control samples are kept under similar laboratory conditions (usually in close proximity to the experimental samples) without being exposed to the intervention (to serve as a negative control) or are being exposed to another intervention that has an established effect (to serve as a positive control). The negative control is typically used to establish the baseline for the intervention of interest's effect and the positive control usually is used to set a ceiling for the effect or is used for comparison of efficacy. Therefore, the observed effect in experimental samples is always being compared to control samples. The effect is measured after adjustments for controls, and data is typically interpreted in light of statistical calculations. If we assume that NCDCI can modify the behavior of distant cells, can this phenomenon confound the intervention effect in *in-vitro* experiments? In other words, can we have a placebo-like effect in cellular or biochemical experiments? If NCDCI result in an increase in positive effects in the negative control group or a decrease in the positive effects in the intervention and/or positive control groups, then this would confound the experimental results. A closer look at the design of a few experiments with NCDCI (Kaznacheev et al., 1980; Farhadi et al., 2007; Rossi et al., 2011; Chaban et al., 2013) shows that in these studies, as mentioned above, there are two sets of negative controls: namely the detector group and the non-detector control group (Farhadi et al., 2007). These studies showed that there is a significant difference in the magnitude of the effects between the detector group and the non-detector control group. Unfortunately, the limited number of these studies to date does not allow us to determine (i) which types of experiments are more prone to NCDCI effects, (ii) whether this phenomenon only affects experiments with live cells (i.e., those in cell biology), or (iii) whether it can also be seen in biological studies that involve biochemistry, molecular biology, and plant and animal studies. Such questions need to be addressed in future studies.

A better understanding of NCDCI can help us recognize whether the observed effects on the controls that we use to denote the baseline of our experiments is related to this phenomenon. The knowledge that can be obtained from further exploration of this field is not limited to detecting its ability to manipulate baseline effects in biological experiments. It could also result in a better understanding of cell physiology and might provide a way to use this novel intercellular communication system for detecting and ultimately controlling cell behavior and function. Given the current state of technology, there is no practical method to detect, record or reproduce the communication signals responsible for NCDCI. We need a much better understanding of these cellular interactions using highly sensitive detectors and computer-assisted pattern analysis. It is my belief that in the near future, this new information will introduce a revolutionary mechanism to the field of cellular biology.

### Conflict of interest statement

The author declares that the research was conducted in the absence of any commercial or financial relationships that could be construed as a potential conflict of interest.
